# Shallow deformation of the San Andreas fault 5 years following the 2004 Parkfield earthquake (Mw6) combining ERS2 and Envisat InSAR

**DOI:** 10.1038/s41598-018-24447-3

**Published:** 2018-04-16

**Authors:** Guillaume Bacques, Marcello de Michele, Daniel Raucoules, Hideo Aochi, Frédérique Rolandone

**Affiliations:** 1 BRGM, Orléans, France; 2 ISTEP, CNRS UMR 7193, Université Pierre et Marie Curie, Paris, France

## Abstract

This study focuses on the shallow deformation that occurred during the 5 years following the Parkfield earthquake (28/09/2004, Mw 6, San Andreas Fault, California). We use Synthetic Aperture Radar interferometry (InSAR) to provide precise measurements of transient deformations after the Parkfield earthquake between 2005 and 2010. We propose a method to combine both ERS2 and ENVISAT interferograms to increase the temporal data sampling. Firstly, we combine 5 years of available Synthetic Aperture Radar (SAR) acquisitions including both ERS-2 and Envisat. Secondly, we stack selected interferograms (both from ERS2 and Envisat) for measuring the temporal evolution of the ground velocities at given time intervals. Thanks to its high spatial resolution, InSAR could provide new insights on the surface fault motion behavior over the 5 years following the Parkfield earthquake. As a complement to previous studies in this area, our results suggest that shallow transient deformations affected the Creeping-Parkfield-Cholame sections of the San Andreas Fault after the 2004 Mw6 Parkfield earthquake.

## Introduction

**Figure 1:**
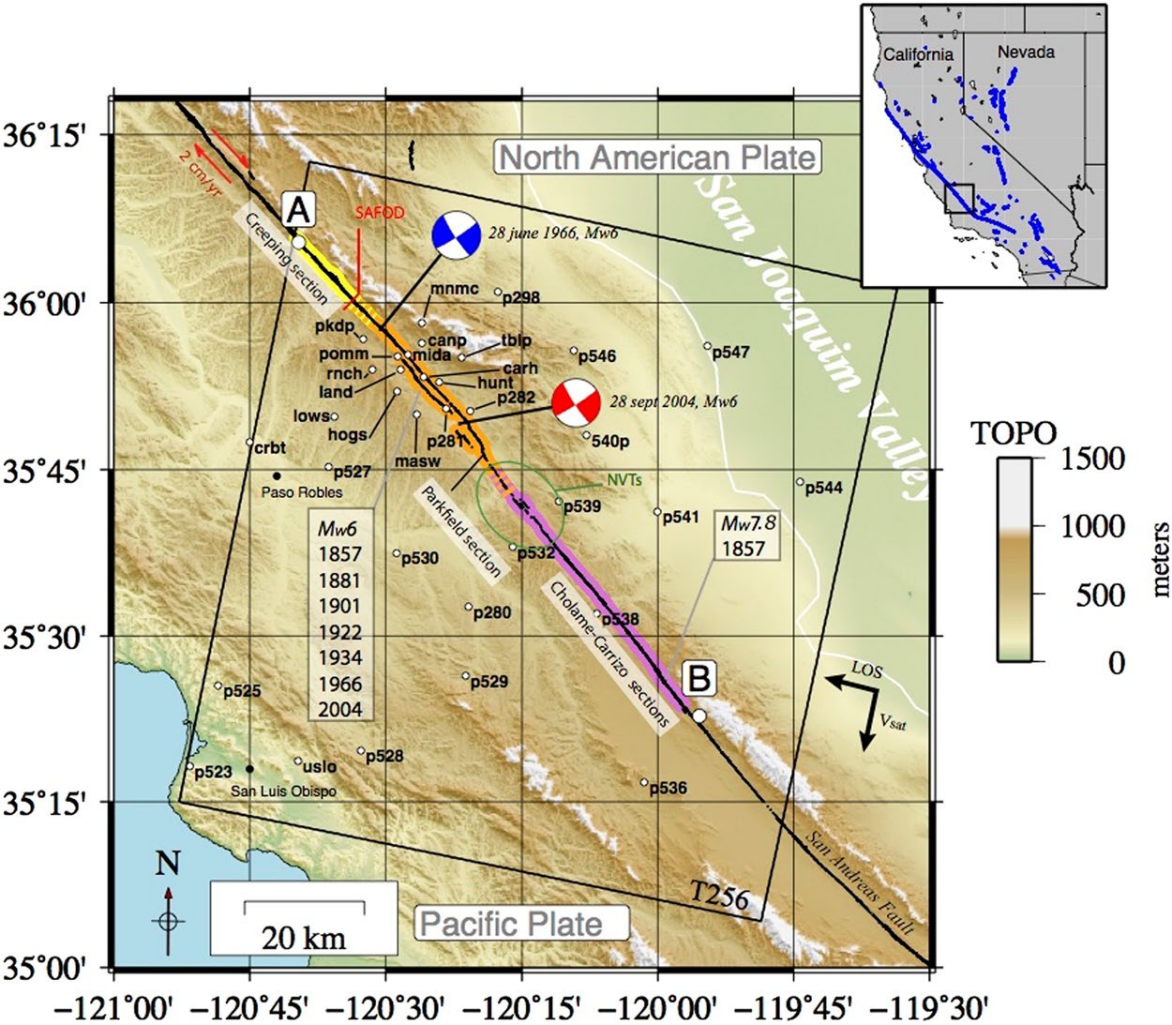
Location map and acquisition geometry (T256) of the Parkfield section (orange), Cholame-Carrizo section (purple) and the Creeping section (yellow). Position and name of the GPS stations of the USGS-Central California Network are presented. The 2004 and the 1966 Mw6 earthquakes epicenters and focal mechanisms associated are presented in red and blue respectively. The topography is derived from the SRTM. The location of the San Andreas Fault Observatory at Depth is also presented (SAFOD). Note: only the San Andreas Fault is presented here. Figure generated with Generic Mapping Tools (GMT 5.1.2, http://gmt.soest.hawaii.edu^35^).

The Parkfield section of the San Andreas Fault (SAF) in California (USA) is a 25 km long dextral strike-slip fault lying at the boundaries between two main sections of the San Andreas Fault in California: the central section at its Northwestern limit and the Cholame-Carrizo section at its southeastern limit (Fig. 1). It is considered a transitional zone between two contrasting mechanical behaviors (aseismic versus seismic). While the Central section is known to stably creep at a rate of 2–3 cm/yr^1–5^, later referenced as the Creeping section, the Cholame-Carrizo section, later referenced as the Cholame section, is locked since 1857 (Fort Tejon earthquake, Mw 7.8)^6–8^. Since 1857, 6 quasi-similar Mw6 earthquakes have been recorded^9,10^ at the Parkfield section, in 1857, 1881, 1901, 1922, 1934, 1966, and 2004. Until the earthquake of 28 September, 2004 (PKEQ), a mean recurrence time of 22 ± 5 years was proposed^9,10^. However, the PKEQ, 15 years delayed from the initial forecast, revealed that the recurrence time could be more variable than expected from earlier studies. Synthetic Aperture Radar interferometry (InSAR), in complement to field instrumentation, has provided evidence of transient deformations along the Parkfield section of the SAF in relation to the PKEQ that are suspected to play a key role in the seismic cycle^11,12^. In this work, we concentrate on the near field of the fault trace. We aim to provide high spatio-temporal InSAR measurements after the PKEQ, from 2005 to 2010, to better constrain the spatio-temporal evolution of the fault displacement at the surface along the Parkfield section of the SAF.

## Transient Deformations at Parkfield Section

During the time period from 1992–2004, the creep rate spatially decreased along the SAF: from about 2 cm/yr at the Creeping section, to 1.4 ± 0.3 cm/yr at the Northwestern Parkfield section, to 0.6 ± 0.3 cm/yr at the southeastern Parkfield section, and finally to ~0 cm/yr at the Cholame-Carrizo section^2,3,11^. Additionally, two–colored electronic distance meters and InSAR time series analysis have revealed a temporal change of the creep rate along the Parkfield section during the same time period (notably an episodic creep acceleration between 1999 and 2000 at the location of the PKEQ epicenter^11^). These transients creep indicate that the Parkfield section experienced an irregular spatio-temporal strain release during the last decade preceding the PKEQ^11,13^.

The post-seismic relaxation following the PKEQ has been documented through the use of global positioning systems (GPS), InSAR, and seismological analyses. The seismicity distribution indicates that the post-seismic relaxation affects a larger area than the co-seismic rupture trace, including the Creeping and Cholame sections. Turner *et al*.^14^ noticed a relative increasing rate of micro-seismicity (about 20%) between 2004 and 2007 with respect to the mean rate calculated over the period from 1986–2011 along the first 20 km of the Creeping section starting from the 1966 Parkfield Mw 6 epicenter (Fig. 1). This is often associated with an increase of the creep rate at depth^15,16^. In addition, the PKEQ has triggered non-volcanic tremors (NVTs, Fig. 1) at the transition between the Parkfield and Cholame sections^17–19^, which are associated with slow slip events occurring at 20 km depth^17^, or viscoelastic relaxation of the lower crust in response to the PKEQ^18^. These studies show that the PKEQ induced long-term perturbations of crustal properties in the SAF zone at depth^17–20^.

Geodetic studies also suggest that, following the PKEQ, the Parkfield section exhibits a complex spatio-temporal behavior, including both after-slip mechanisms at shallow depths (between 0 and 20 km) and viscoelastic relaxation at larger depths (more than 20 km). Moreover, the equivalent seismic moment released by these mechanisms over the 5 years following the PKEQ is more than twice the one released during the PKEQ^21–25^. From the comparison between the average fault slip rate before and after the PKEQ derived from InSAR and GPS (between the 1986–2004 and 2006–2011 periods) and seconded with numerical models, Barbot *et al*.^12^ detected the presence of creep at depth south of the PKEQ epicenter at the transition between the Parkfield and the Cholame sections, which might indicate the presence of a soft barrier at depth, acting as an efficient arrest to earthquake ruptures for the Mw6 earthquakes but allowing propagation of larger ruptures.

Significant insights into the fault properties have been provided from the analysis of the spatio-temporal evolution of the transient deformations along the Creeping-Parkfield-Cholame sections. In complement to previous studies in this area, here we intend to better characterize the spatio-temporal distribution of the shallow fault displacement field after the PKEQ between 2005 and 2010.

## Methods and Data Processing

**Figure 2:**
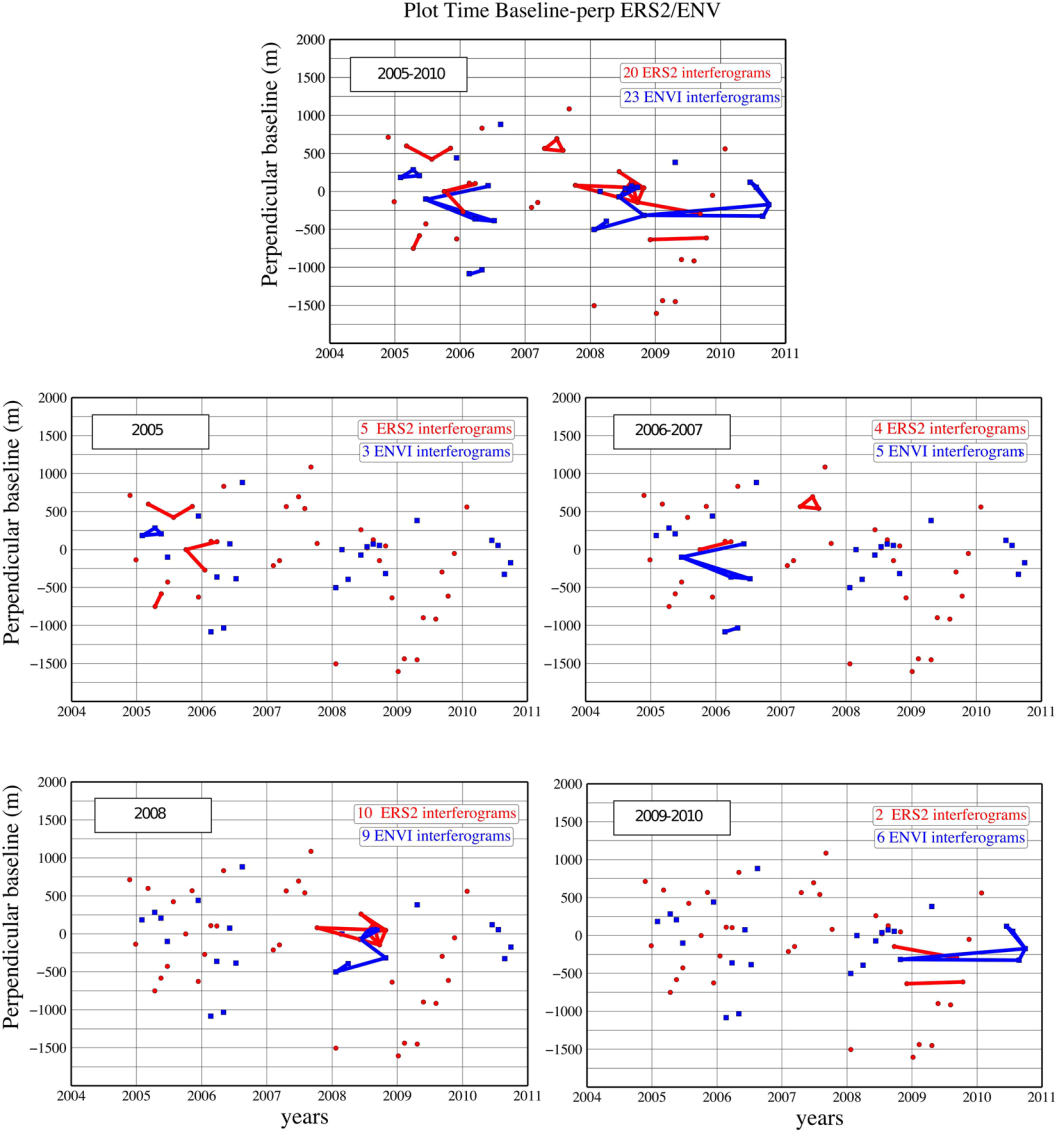
Perpendicular baseline as a function of time for each periods (2005–2010, 2005, 2006–2007, 2008, 2009–2010). Blue dots and red dots represent Envisat and ERS2 acquisitions respectively. Blue lines and red lines represent Envisat and ERS2 interferograms respectively. Figure generated with Generic Mapping Tools (GMT 5.1.2, http://gmt.soest.hawaii.edu^35^).

We constructed both ERS2 and Envisat interferograms from raw Synthetic Aperture Radar (SAR) data, aiming at producing mean deformation rates on annual time-spans. Processing is done via the GAMMA Software^26^. We selected the descending track 256 for both ERS2 and Envisat (C-band, i2 mode, polarization VV, frame n°2889, Fig. 1). Barbot *et al*.^12^ processed Envisat data (track 256, frame 2889) to produce a mean deformation velocity map around the Parkfield section between 2006 and 2010. In this study we focus on a longer period (2005–2010) with a larger dataset, including additional Envisat ASAR and ERS2 acquisitions. Our dataset is composed of 37 raw images from ERS2 and 25 raw images from Envisat ASAR (Fig. 2). To increase the temporal data sampling, we stack unwrapped interferograms jointly from ERS2 and Envisat data. Then, we derive the evolution of the along-fault creep distribution per sub-period instead of a full-period times-series analysis, such as the one proposed by de Michele *et al*.^11^. We co-register both ERS2 and Envisat Single Look Complex images upon one unique geometric model. Then we compute ERS2 and Envisat interferograms separately. Once the interferograms are computed and multilooked (2 in range and 10 in azimuth), orbital and topographic fringes are removed using precise orbital information and Shuttle Radar Topography Mission (SRTM) at 30 m resolution per pixel.

Two major biases can affect SAR interferograms when dealing with tectonic analyses. Firstly, non-stationary tropospheric SAR signal delay can add an undesired InSAR signal component. This delay can be correlated to topography, in which case it can be modeled and removed by linear regression with a digital elevation model (SRTM). Data stacking is also an effective way to reduce the non-topography-related atmospheric noise. Secondly, random temporal changes on the surface of the Earth can reduce the signal to noise ratio (snr), which we refer to as SAR signal coherence. Therefore, a spatial filter is applied to the wrapped interferograms to increase the snr^27^ and facilitate the interferogram unwrapping procedure performed using the minimum cost flow algorithm^28,29^. We mitigate the atmospheric bias for each interferogram by estimating a linear phase trend with respect to the elevation model that we remove from each interferogram^30^. We initially calculate about 400 interferograms, most of which present low signal coherence, either due to temporal changes on the surface or to geometric signal decorrelation. As a further step, to avoid the use of low signal coherence interferograms, we perform a visual inspection. Among the total number of computed interferograms, we select 43 coherent interferograms that we use for further analyses. This significant loss of data is mainly due to poor signal coherence, mainly generated by random temporal changes in the vegetation cover, and/or persistent widespread atmospheric bias, notably at the location of the Creeping section area.

We derive the spatio-temporal evolution of the ground velocity distribution along the fault trace by stacking unwrapped interferograms from both ERS2 and Envisat. We define 5 periods of interest depending on the data availabilities (Fig. 2). We present 5 different data stacks. We calculate: (1) the whole study period from 2005 to 2010 (43 interferograms, 20 ERS2 - 23 Envisat); (2) the year 2005 (8 interferograms, 5 ERS2 - 3 Envisat); (3) the period from 2006–2007 (9 interferograms, 4 ERS2 - 5 Envisat); (4) the year 2008 (19 interferograms, 10 ERS2 - 9 Envisat); (5) the period from 2009–2010 (8 interferograms, 2 ERS2 - 6 Envisat). Although the temporal distribution of the interferograms does not perfectly match the time intervals as previously defined, each sub-period contains enough different sets of independent interferograms. As an exception, the 2005 and the 2006–2007 periods contain 1 common ERS2 interferogram, for the sake of snr improvement. This processing allowed us to derive the time evolution of ground deformation in the near field of the fault between 2005 and 2010, per sub-periods. The stacking method is performed in radar geometry. The results are then geo-referenced. Afterwards, we fit the InSAR signal to the GPS reference frame. The InSAR-GPS ramps in each stack are fixed by removing a ramp model built from the difference between InSAR values in line of sight geometry (LOS) and GPS velocities (Central California GPS database, USGS) converted from north-east-up components (ITRF2008) to LOS (L_north = 0.092, L_east = −0.379, L_up = −0.921).

We use the COSI-Corr^31^ «Stacking Profile tool» to extract the velocity offsets across the fault from the stacked interferograms. The COSI-Corr Stacking Profile tool performs a linear regression on the InSAR stack profiles at each side of the fault trace, yielding one offset value at each measured location. We took measurements at less than 1 km apart from the fault trace all along the fault line as suggested in de Michele *et al*.^11^ for this specific area (~2 km across fault baseline equivalent). The ground velocity offsets across the fault are then projected along strike and compared with creep-meter values over the same time period (available from USGS website). Creep-meter devices consist of two piers spaced 30–60 m apart and located on opposite sides of the fault connected to each other by a wire^1,32^. The cumulative horizontal along strike displacement is derived from the time evolution of the angle of the wire from the strike of the fault. From this cumulative displacement, we extracted creep rate per sub-period (the same sub periods as the interferogram stacks). As a simplification, we assume that the fault displacement at less than 1 km from the fault line is mainly due to pure strike-slip displacement. This assumption is roughly corroborated from a preliminary analysis of the available GPS time series. The velocity distribution along the fault derived from InSAR is converted from rad/yr (LOS) to cm/yr (strike-slip dextral motion). This assumption allows us to make the comparison between InSAR and creep-meters easier.

**Figure 3:**
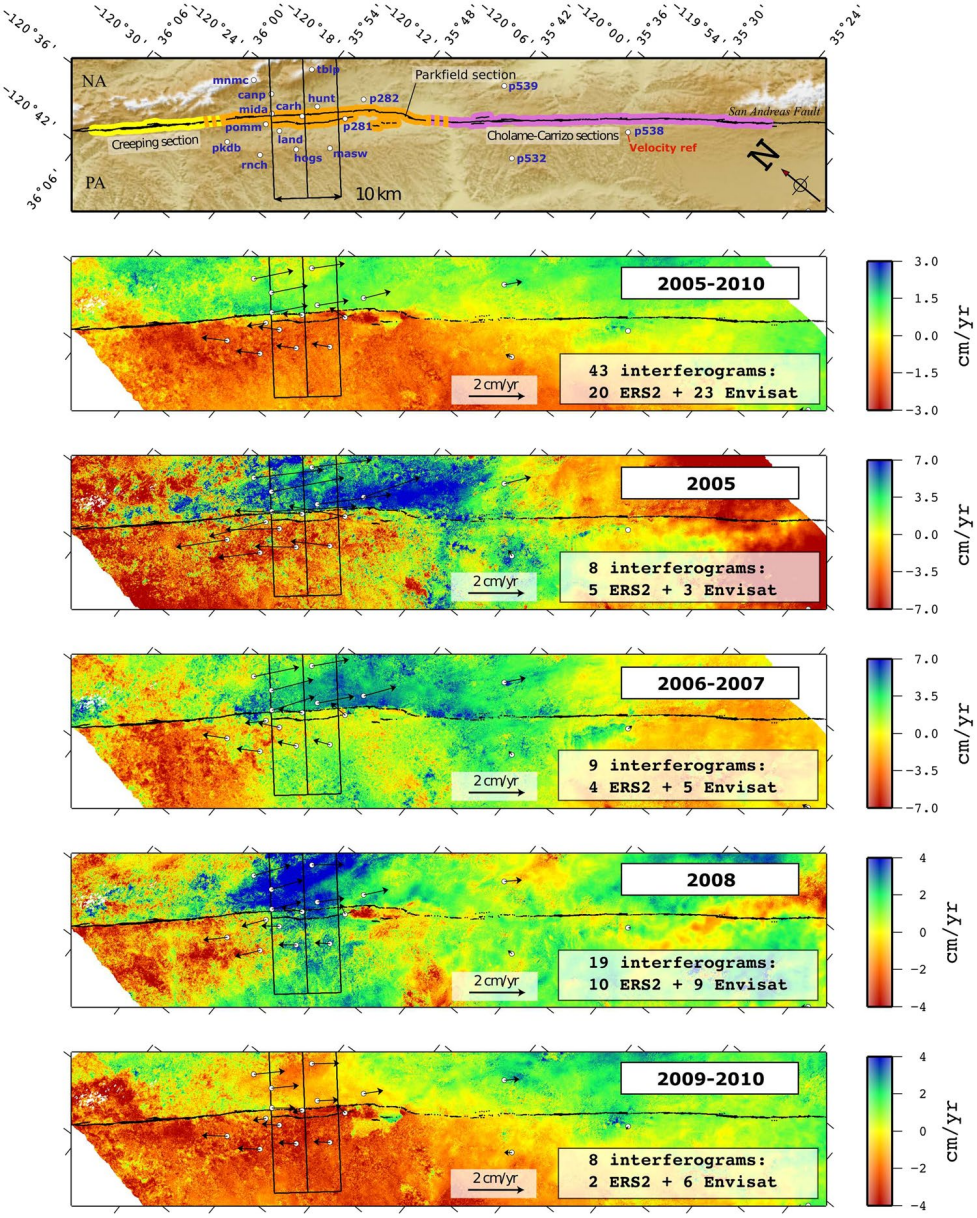
Stacked interferograms according to the 5 periods between 2005 and 2010. PA and NA reference the Pacific Plate and the North American plate respectively. Velocity fields are presented in cm/yr in strike-slip geometry. Top figure represents the SAF geometry with the Creeping section (yellow), the Parkfield section (orange) and the Cholame-Carrizo section (purple). The black inner rectangle indicates the area of profiles in Fig. 4. The location of permanent GPS stations from the Central California Network with the horizontal velocity vectors are also presented. The GPS and InSAR reference velocity if the P538 GPS station. To avoid data saturation, the color scale bars are adapted accordingly to the data range variability.

As a final step, all data are referenced to station P538 (set to 0 cm/yr), as P538 station is both close to the fault line and steady through time (see top picture, Fig. 3).

## Results

### SNR analysis (Stacks overview)

The 5 stacks are presented in Fig. 3. For the period of 2005–2010, ERS2 and Envisat stack shows a clear bimodal velocity distribution, indicating dextral shear between the North American plate and Pacific plate as expected in this area. However, this bimodal velocity distribution is more roughly observable for all sub-period (i.e. sub-periods 2005, 2006–2007, 2008, and 2009–2010). Indeed, we highlight that some sub-period stacks are more affected by atmospheric bias and noise because they are constructed from a lower number of interferograms. This can be seen around the westward limit of the Creeping section (see Fig. 3). Nonetheless, the local offset measured in the near field of the fault should still be considered as an interpretable measure. This is because, while InSAR local signal coherence might affect the precision on the offset measurements, we can exclude atmospheric biases at this very local scale. We report that, the 2009–2010 stack contains an unwrapping error at the location of the 2004 earthquake epicenter. Therefore, interpretations at this specific location shall be taken carefully.

**Figure 4:**
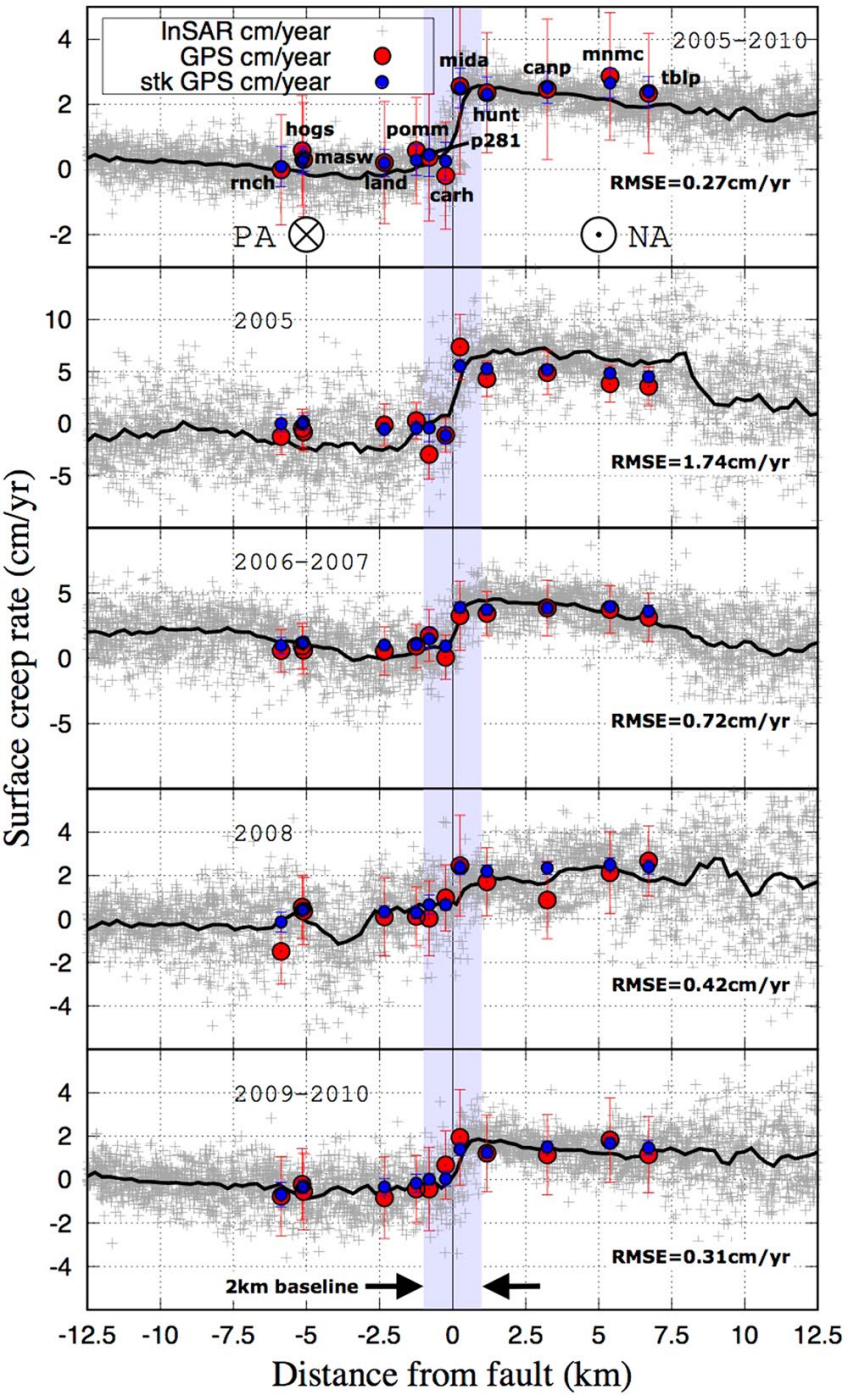
Across fault profiles, 25 km long and 10 km width, derived from the stacked interferograms (Fig. 3, black rectangle) for each periods and comparison with the GPS data. PA and NA reference the Pacific Plate and the North American plate respectively. Grey dots represent InSAR values in fault strike geometry and black lines their spline interpolation. The red dots present the GPS velocities converted from north-east-up components to LOS and to fault strike geometry in order to allow reliable comparison with InSAR derived estimations. Blue dots present the GPS horizontal fault strike component (case 2). The Root Mean Square Error (RMSE) is estimated between InSAR and GPS (case 2). To avoid data saturation, the vertical scales were adapted accordingly to the data range variability.

To further investigate the InSAR data reliability and evaluate our pure horizontal fault motion hypothesis mentioned above, we compare InSAR data and GPS for each period using a profile across the Parkfield section, more densely covered by GPS stations (Figs 3 and 4). Firstly, the GPS velocities are projected on the LOS and then converted in strike-slip geometry following the same procedure applied to our InSAR data (case-1, red circles, Fig. 4). Secondly, as a proxy to evaluate the pure strike fault motion hypothesis, we compare the InSAR in strike-slip geometry with the fault strike component of the GPS horizontal velocities (case-2, blue circles, Fig. 4). Qualitatively, from the visual comparison between InSAR and GPS (Fig. 4) one can observe the good fit between the two, especially for the 2005–2010 period. Nonetheless, some differences between GPS case-1 and case-2 can be noticed in 2005 at the location of MIDA station (Fig. 4). We speculate that this difference might be due to the vertical or horizontal fault-normal components of the ground deformation that affects our fault motion hypothesis at this location in 2005. However, we do not observe other significant differences between the GPS (red dots) and the pure along strike fault motion derived from the GPS (blue dots) for other sub-periods. Additionally, from the analysis of the root mean square error (rmse) between the InSAR profiles in Fig. 4 and the GPS strike component velocities (blue dots), we observe a good agreement between the two. Precisely, we assessed a rmse of 0.27 cm/yr for the 2005–2010 period; 1.74 cm/yr for the 2005 period; 0.72 cm/yr for the 2006–2007 period; 0.42 cm/yr for the 2008 period; and 0.31 cm/yr for the 2009–2010 period, which, compared to the total amount of near field surface offset measured per sub-period (Fig. 4), might be considered a good score.

**Figure 5:**
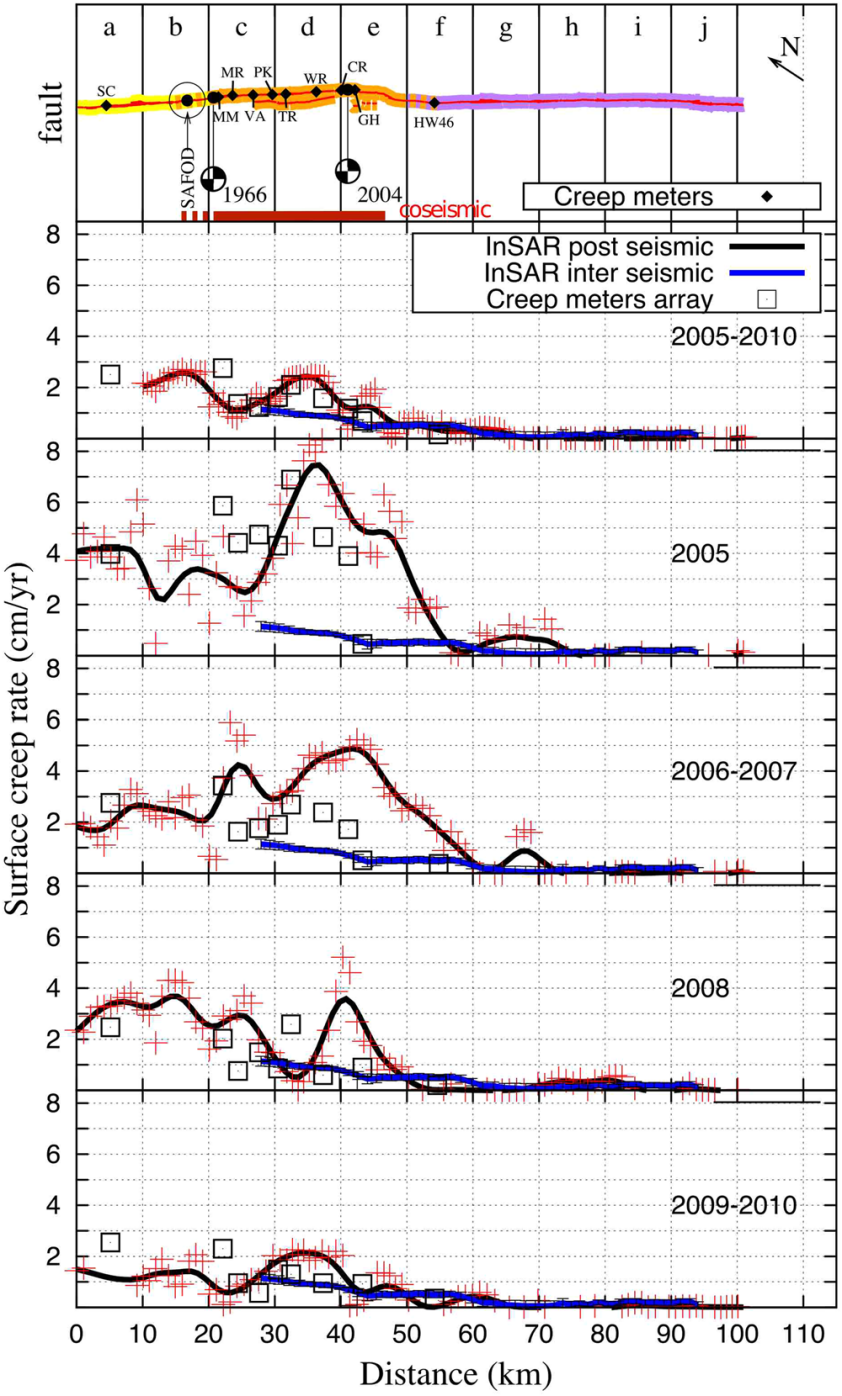
Along fault surface creep rate for each periods derived from InSAR (red cross) with spline interpolation (black lines) and comparison with creep-meters white (squares) and interseismic creep rate (blue lines) extracted by de Michele *et al*.^11^ between 1993 and 2004. The top figure presents the fault geometry (yellow: Creeping section, orange: Parkfield section, purple: Cholame-Carrizo section) with creep meters positions and names: Slack-Canyon (SC), Middle-Montain (MM), Middle-Ridge (MR), Varian (VA), Parkfield (PK), Taylor-Ranch (TR), Work-Ranch (WR), Carr-Ranch (CR), Gold-Hill (GH), HighWay-46 (HW46). The 2004 coseismic surface rupture length is indicated following Rymer *et al*.^33^. The location of the San Andreas Fault Observatory at Depth is also presented (SAFOD).

The high spatial resolution (about 50 m per pixel) allows us to derive a detailed along fault evolution of the surface creep rate (Fig. 5). The across-fault offsets (within 1 km of the fault) extracted from each sub-period stack are reported along the fault, according to the distance from A to B (Fig. 5). For each sub-period, we compare each InSAR profile to the creep-meter rate and to the 1992–2004 inter-seismic along-fault velocity profile derived by de Michele *et al*.^11^ (blue line). From the comparison between the spline interpolation (black line) and the measured offset (red cross) we can assess the snr of the systematic velocity offset extraction procedure that we used. While we observe a good snr between the offset measurements with respect to the spline interpolation for the 2005–2010, 2006–2007, 2008, and 2009–2010 periods, the 2005 period seems to be affected by a lower snr. However, we observe that all the velocity profiles spatially mimic the broad SAF segmentation at the Parkfield (i.e. transitional creeping behavior) and Cholame (i.e. locked behavior) sections.

**Figure 6:**
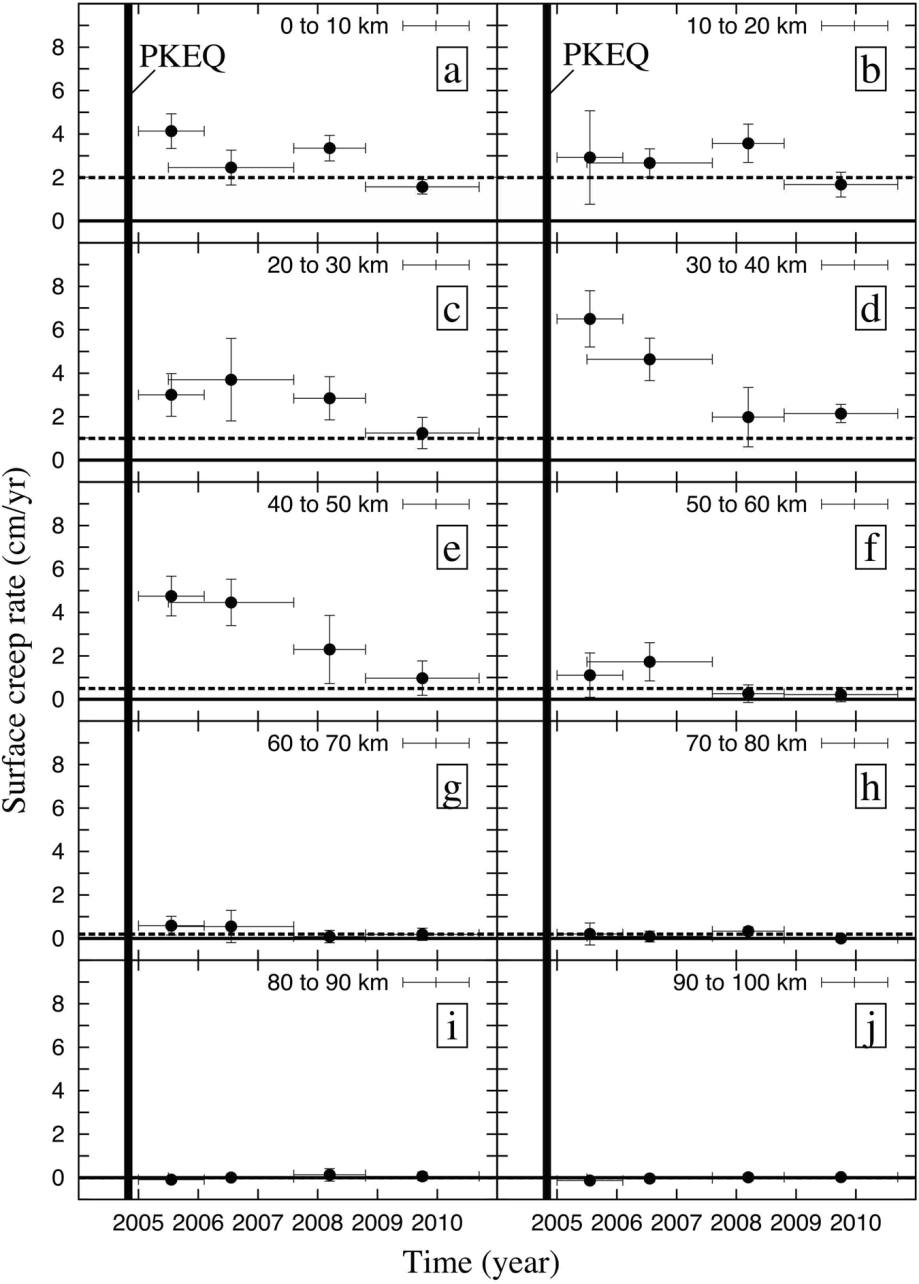
Time evolution of the shallow creep rate along the Creeping, Parkfield, Cholame sections (cm/yr). The Four periods (2005, 2006–2007, 2008, 2009–2010) are represented as the time error bars. The mean creep velocity is assessed every 10 km from the InSAR along fault creep rate profils (see Fig. 5) and the standard deviation is presented as vertical error bars. The location of each plot is referenced by a letter from « a » to « j » according to the top picture Fig. 5. The interseimic mean creep is indicated by the top edge of the grey rectangles (values derived from Ryder *et al*.^3^ and de Michele *et al*.^11^). The bold dark vertical line symbolizes the date of the PKEQ.

Finally, to better visualize the creep evolution with time, we split the fault trace into 10 subsections, each 10 km long (Fig. 6). We referenced them from “a” to “j” along the Creeping, Parkfield, and Cholame sections of the SAF. For each subsection and sub-period, we focus our attention on the comparison between the mean creep rate evolution with respect to the inter-seismic references from Ryder *et al*.^3^ for the Creeping section and from de Michele *et al*.^11^ for the Parkfield and Cholame sections. As shown in Figs 5 and 6, the surface creep rate along the three sections of the SAF exhibits a complex spatio-temporal evolution following the PKEQ.

## Observations for each sub-period and sub-sections

### The Creeping section

Northwest of the Parkfield section, the 20 km of the Creeping section included in our analysis experienced an irregular evolution of the surface creep rate following the PKEQ (Figs 5 and 6ab). In 2005, the surface creep rate reached about 4 ± 1 cm/yr at Slack-Canyon, which is twice the inter-seismic reference values^11^. Then it decelerates in 2006–2007 to reach the inter-seismic values (about 2 ± 1 cm/yr at Slack-Canyon). After an acceleration period in 2008 (about 3 ± 0.5 cm/yr at Slack-Canyon), it falls to the inter-seismic reference values in 2009–2010 (about 1.5 ± 0.5 cm/yr). Since the 2009–2010 stack is affected by the lower snr at this location, the creep velocity might be under-estimated. This irregular spatio-temporal evolution of the shallow creep is likely due to the stress field perturbation induced by the PKEQ propagating towards the Creeping section.

### The Parkfield section

Following the PKEQ, the Parkfield section experienced a gradual spatio-temporal decrease in creep rate more typical of a post-seismic relaxation (Figs 5 and 6c,d,e). The highest creep perturbation occurs in 2005, where the creep rate reaches about 7 ± 1.5 cm/yr at the Taylor-Ranch location. This is the highest value that we have measured in this study, consistent with creep-meter measurements at this location. We estimate a mean creep rate during 2005. However, creep at Taylor-Ranch could have been higher in the earlier stage of the post-seismic period starting at the end of 2004, as suggested by Johanson *et al*.^21^. Creep gradually decelerates to 5 ± 1 cm/yr in 2006–2007, 2.5 ± 0.5 cm/yr in 2008, and finally 2 ± 0.5 cm/yr in 2009–2010. The spatial distribution of surface creep is not homogeneous all along the length of the Parkfield section (Fig. 5). The central part of the Parkfield section presents the highest variation in amplitude ranging from 7 ± 1.5 cm/yr in 2005 to 2 ± 0.5 cm/yr in 2009–2010. The Northwestern part shows creep rate values similar to the ones measured in the Creeping section. Moreover, during 2008, we observe important lateral variations in the creep rate distribution between Middle-Mountain and Gold-Hill (Fig. 5).

### The Cholame section

In 2005 at the Parkfield-Cholame transition section, surface creep sharply decreases from 5 ± 1.2 cm/yr at the Gold-Hill location to ± 0.5 cm/yr at km 60 (Fig. 5). We observe a similar behavior in 2006–2007 (5 ± 0.5 cm/yr at Gold Hill, 1 ± 0.5 cm/yr at kilometer 60), but the transition zone seems to extend a few km southeastward compared to 2005 (Fig. 5). We infer that the surface creep has decayed toward the Cholame section likely due to the presence of a stronger section of the fault or a change of fault friction properties between the Parkfield and Cholame sections. The Cholame section shows shallow creep in 2005 and 2006–2007 (Fig. 6f,g) along its first 20 km from the 2004 PEQ epicenter. Creep accelerated up to 2 ± 0.5 cm/yr during the 2005 and 2006–2007 periods, before falling to the inter-seismic reference values in 2008 (about ± 0.2 cm/yr). Except for the presence of a burst of creep of about 1 ± 0.2 cm/yr, localized at km 65 in 2005 and 2006–2007 (Figs 5 and 6g), we do not detect any surface creep signature far from km 70 along the fault during the study period.

## Discussion

From the combined stacks of ERS and Envisat SAR interferograms, we measure the transient surface creep at the Creeping, Parkfield, and Cholame sections of the SAF during the 5 years following the 2004 earthquake. Based on previous studies, such transients in the surface creep signal were not detected during the 10 years prior to the PKEQ. These observed perturbations are characterized by both high spatial variability (with large creep values up to ~7 ± 1.5 cm/yr in 2005) and a complex temporal evolution with oscillatory like behavior. They extend from Slack-Canyon (Creeping section) to the first 10 km of the Cholame section (Fig. 5a–g), making the total portion of the SAF perturbed after the PKEQ about twice the fault rupture length reported by Rymer *et al*.^33^ for the coseismic. This observation is in agreement with the work of Turner *et al*.^14^, who state that the PKEQ could have affected the Creeping section further northwest, resizing the total affected fault length at depth. Additionally, the surface creep along the first 20 km from the PKEQ epicenter toward the Cholame section in 2005 and 2006–2007 detected in this study might corroborate the presence of a soft barrier at the Parkfield-Cholame transition zone^12^.

We speculate that this oscillatory spatio-temporal behavior of the SAF might be due to a complex combination of PKEQ postseismic lower crust relaxation mechanisms in addition to the rate-and-state frictional fault behavior in response to the local or distant tectonic and/or non-tectonic sources of stress-change that radiate along the SAF. However, the relation between the shallow fault motion and deep fault motion is not yet clear and it still represents a scientific debate. The geological and structural complexity of the shallow depth crust shall be the set of additional phenomena that should be investigated further before any conclusion can be made on the fault properties from these data. Notably, we do not observe, for the central Parkfield segment, a convergence between the creep measurements of this study and the mean creep rate distribution documented by de Michele *et al*.^11^, even in 2009–2010, almost 6 years after the PKEQ. It is difficult to conclude if that is due to a long term change of the fault frictional properties or if this lasting transient creep is just part of more complex seismic cycle strain loading/release processes not yet fully documented. There is the possibility that these shallow transients along the analyzed segments might be somehow related to the fault at depth. These relations should be investigated in further studies. Nonetheless, the mapping of the transient deformations on the SAF along these three sections might bring constraints on the charge-discharge cycle of the fault system and perhaps give some hints to the understanding of the Parkfield earthquake sequence variability.

Creep meter arrays initially appear to be good candidates for such monitoring, as they present both a long time recording (from the 60 s to now) and high sampling rate of the ground deformation. However, while we observe that the midterm spatio-temporal trend between InSAR and the creep meters is similar (see Fig. 5, 2005–2010 period), when it comes to short term measurements, creep meter estimates might become less reliable in terms of fault motion. As shown in the San Francisco Bay area faults, creep meters might undergo short term rate changes due to various sources of environmental changes^34^. We also speculate that this difference might be exacerbated by the difference between the creep-meter across fault baseline scale (~60 m) and the ~2 km across fault baseline in InSAR (see Fig. 4). In other words, despite the ability of creep-meter devices to sample the fault creep rate with a high frequency, they are limited to fully constraining the amplitude of the surface creep rate for short periods and for small across fault deformation gradients compared to the InSAR or GPS techniques. The use of the combined present day SAR missions, such as the Sentinels 1A/1B (and future missions such as the Sentinels 1D and 1E), thanks to their high revisit time and large spatial coverage, might fulfill the monitoring needs as a complement to ground measurements from field investigations and permanent GPS stations.

In this study we made the hypothesis of purely strike slip horizontal fault motion to favor the comparison between InSAR and the creep-meter array. This hypothesis is corroborated by the good agreement between fault strike GPS velocities components and InSAR data. However, previous GPS based studies over the central portion of the SAF highlighted complex 3 dimensional deformations patterns around the fault^2^. Consequently, this hypothesis needs to be verified further, maybe using recent InSAR based technics, such as Multiple Aperture Interferometry, and combining ascendant descendent InSAR acquisitions.

## Conclusions

By combining ERS2 and Envisat interferograms, we could improve the spatio-temporal sampling of the surface creep evolution for the Creeping-Parkfield-Cholame sections of the SAF, between 2005 and 2010. This methodological improvement, along with the high spatial resolution of the SAR interferograms, allowed us to estimate a map of the near field SAF surface displacement after the PKEQ. These measurements suggest the presence of transient surface deformations on this complex section of the SAF that contrast both in amplitude and distribution with what was documented during the 10 years prior to the PKEQ with InSAR. We observe that, after the PKEQ, the shallow creep rate on the Creeping, Parkfield, and Cholame sections has been highly affected from 2005 to 2010, with surface creep values reaching up to 7 ± 1.5 cm/yr with an oscillatory like spatio-temporal evolution that reveal a complex and not steady behavior of the SAF on the Creeping, Parkfield, and Cholame sections. In these terms, the measurement of the near field surface fault motion with a high spatio-temporal resolution appears as the first step in the improvement of our understanding of the geophysical process driving the surface faults kinematics behavior. This leads to the fact that monitoring both the long-term shallow fault motion and transient surface creep rate all along the Creeping, Parkfield, and Cholame sections still presents a primary scientific interest. In these terms, future geodetic based studies in this area shall benefit from the use of combined present day SAR missions, such as the European Sentinels 1A/1B missions (and future missions such as the Sentinels 1D and 1E).
